# Resistance to anti-TB drugs: evaluation of universal resistance surveillance implementation in Chile

**DOI:** 10.5588/ijtldopen.25.0507

**Published:** 2026-06-15

**Authors:** G.N. Barra, M. Oyarte, K.K. Ivani, Á.D. Briceño, I.M. Espinoza, T.L. Calderón, M.M. Juica, A.S. Bordón, J.F. Oviedo, F.A. Muñoz

**Affiliations:** 1 Departamento Biomédico Nacional y de Referencia, Instituto de Salud Pública de Chile, Unidad de Investigación EInnovación; 2 Sección Micobacterias, Departamento Biomédico Nacional y de Referencia, Instituto de Salud Pública de Chile, Santiago, Chile.

**Keywords:** tuberculosis, drug susceptibility testing, line probe assay, MDR-TB, epidemiological surveillance

## Abstract

**BACKGROUND:**

TB remains a public health challenge in Chile, where all bacteriologically confirmed TB cases are tested for drug susceptibility at the National Reference Laboratory of the Instituto de Salud Pública (ISP).

**OBJECTIVE:**

We evaluated the impact of implementing universal surveillance for rifampicin and isoniazid resistance in Chile and described resistance profiles and epidemiological factors.

**DESIGN:**

Retrospective analysis of drug susceptibility testing (DST) results from *Mycobacterium tuberculosis* cultures processed at the ISP between 2003 and 2020. DST was performed using line probe assays and mycobacteria growth indicator tube liquid culture. Demographic and clinical data were analysed, and a negative binomial regression model assessed changes before and after universal surveillance in 2014 (2003–2014 vs. 2015–2020).

**RESULTS:**

Among 44,800 isolates analysed, 911 had first-line resistance to isoniazid or rifampicin: 377 showed isoniazid monoresistance, 155 rifampicin monoresistance, 378 multidrug resistance, and one extensively drug-resistant case. Detection of resistant cases increased after universal surveillance (incidence rate ratio = 3.592, *P* < 0.001). Resistance was most frequent in men aged 16–64 and in the central macrozone.

**CONCLUSION:**

Universal surveillance improved detection of drug-resistant TB in Chile, supporting its value for timely treatment adaptation and targeted public health strategies, especially in high-risk demographic and geographic groups.

TB is caused by *Mycobacterium tuberculosis* (MTB), a slow-growing aerobic bacillus transmitted through airborne droplets when an infected individual coughs or sneezes.^1–3^ TB affects the lungs and remains a leading cause of death worldwide.^1–4^ In Chile, TB control efforts were formalised through the National Program for TB Control and Elimination (PROCET).^5^ Diagnosis relies on smear microscopy (BK), culture, and real-time PCR (GeneXpert) for rapid detection of MTB complex and rifampicin resistance.^6^ GeneXpert is recommended for initial diagnosis, whereas BK and culture are essential for treatment monitoring because molecular tests cannot determine viability.^7^

TB treatment involves a two-phase regimen including isoniazid (INH), rifampicin (RIF), pyrazinamide (Z), and ethambutol (E). The initial phase targets metabolically active bacilli and the continuation phase targets slow-growing bacilli in intracellular or caseous environments.^7^ Drug resistance arises primarily from chromosomal mutations, leading to primary (transmitted) or acquired resistance, especially after inadequate or incomplete treatment.^8^ Because multidrug resistance (MDR) is defined by combined INH and RIF resistance, which are the mainstays of first-line treatment, delayed identification compromises treatment effectiveness, prolongs infectiousness, and worsens outcomes.

In Chile, all bacteriologically confirmed pulmonary TB cases are tested for susceptibility to INH and RIF using line probe assays (LPAs) at the National Reference Laboratory (ISP). Since 2014, this policy replaced selective testing and reduced underreporting of resistance, in line with WHO’s End TB recommendations on universal drug-resistance surveillance, providing a clear pre/post-implementation framework (2003–2014 vs. 2015–2020) to assess changes in resistance detection.^6,9^ Resistance to anti-TB drugs remains low compared to other countries in the Americas. In 2019, the estimated incidence of TB was 14.4 cases per 100,000 population.^10,11^ Between 2011 and 2012, a national study reported an overall resistance rate of 8.6% and MDR-TB prevalence of 1.3% in new cases.^12^ Timely identification of INH/RIF resistance strengthens PROCET operations by enabling targeted contact-tracing, rational drug procurement, and accurate surveillance for resource allocation. Across Latin America and the Caribbean, drug-resistance burdens are heterogeneous and higher among previously treated patients. A recent meta-analysis in MDR-TB patients estimated pooled prevalence of pre-extensively drug resistance (pre-XDR) 10% and extensively drug resistance (XDR) 5%, with higher proportions in Peru (pre-XDR 13%, XDR 6%) and Brazil (pre-XDR 16%, XDR 5%), and 4% XDR in Argentina and Colombia.^13^ Haiti reported 7.7% INH resistance among RIF-susceptible cases, underscoring detection gaps beyond RIF testing.^14^ Ecuador, Guatemala, and the Dominican Republic also reported pre-XDR/XDR in 2020, despite limited peer-reviewed studies.^13,15^ In 2022, Bolivia reported a TB incidence of 66 per 100,000, and the PAHO/WHO classifies Bolivia, Haiti, and Peru as ‘endemic’ (100–299 per 100,000 population).^16,17^ This heterogeneity supports sustained universal drug-resistance surveillance.

We assessed the impact of Chile’s universal drug-resistance surveillance by analysing confirmed TB cases from the ISP, comparing data from before and after the 2014 policy implementation, and describing resistance patterns by case category and period.

## METHODS

Before 2011, phenotypic drug susceptibility testing (DST) using the BACTEC MGIT 960 liquid culture system was the routine approach when resistance was suspected or confirmation was required. In 2011, LPA (MTBDRplus v2.0) was implemented at ISP and adopted as the initial test for INH/RIF resistance, with MGIT reserved for confirmation after negative or invalid LPA, clinical discordance, or epidemiological/clinical suspicion. From mid-2014, Chile adopted universal screening for first-line (INH/RIF) resistance for all bacteriologically confirmed TB cases (new and previously treated), under the National Tuberculosis Program technical standards, which govern prevention, detection, diagnosis, treatment, and surveillance.^18,19^

We analysed DST records from bacteriologically confirmed pulmonary TB processed during 2003–2020. The unit of analysis was the treatment episode (episode-based surveillance), defined as treatment initiation or re-initiation. Within each calendar year, one record per episode was analysed (earliest DST), unless the DST profile changed; in that case, each distinct profile was analysed separately. Episodes in subsequent years reflecting re-initiation/retreatment were considered distinct. Inclusion criteria were: i) culture-confirmed MTB complex; ii) first-line resistance testing to INH and/or RIF by LPA and/or MGIT; and iii) valid identifiers enabling record linkage. Exclusion criteria were: non-TB mycobacteria, indeterminate DST after repeat testing, and incomplete identifiers precluding linkage. Treatment regimens followed national standards; however, individual-level regimen assignments were not recorded in the surveillance dataset and therefore not analysed.

### Drug susceptibility testing

Phenotypic DST was performed using mycobacteria growth indicator tube (MGIT 960® system) using the proportion method in liquid medium, following manufacturer guidelines.^20^ The drug panel included streptomycin, isoniazid, rifampicin, ethambutol, pyrazinamide, kanamycin, ethionamide, and moxifloxacin.

### Line probe assay

First-line resistance was assessed with GenoType MTBDRplus V2.0 (LPA), which uses nitrocellulose strips with immobilised probes targeting resistance loci. Strains were inactivated with GenoLyse® (solid and liquid media). PCR amplification, hybridisation, and interpretation of resistance patterns were performed following standard protocols.^21^ Modified Ziehl–Neelsen staining assessed contamination and bacillary presence. Second-line drug-resistance testing (MTBDRsl v2.0) was performed for RIF-resistant/MDR isolates or clinically suspected second-line resistance and was not applied universally.

### Variables

Variable labels were defined and categories were created: sex (female, male); age (≤15, 16–64, ≥65, unknown); macrozone (north, north central, central, south central, south); migration status (foreign-born, Chilean); nationality (foreign-born only): Peru, Haiti, Bolivia, Ecuador, Colombia, Venezuela, other; resistance (INH, MDR, RIF, XDR); treatment history (previously treated, new case); HIV status (positive, not specified). A confirmed HIV-negative category was not systematically captured; therefore, ‘not specified’ was treated as unknown/not reported. HIV status was used only for HIV-specific descriptions/figures and was not included as a covariate in the regression model.

Calendar year was included as a discrete variable and as a categorical period (2003–2008, 2009–2014, and 2015–2020), based on milestones in national diagnostic strategies. These periods represent the pre-LPA phase, a transitional period with greater operational stability, and the implementation of universal screening for first-line drug resistance. To evaluate universal surveillance, results were reported annually and by period; case descriptions were stratified by demographic and clinical characteristics; and rates were computed using population denominators for each stratum.

### Statistical analysis

Stratum-specific rates were calculated using the National Institute of Statistics of Chile (INE) population estimates as denominators.

The relationships between resistance cases (counts), demographic variables, and periods were explored using a negative binomial model of the form:

$$\log\;{\left(\text{cases}\right)}_{i}\;=\;\beta_{0}\;+\;\beta_{1}{\text{Period}}_{i}\;+\;\beta_{2}{\text{Sex}}_{i}\;+\;\beta_{3}{\text{Age}}_{i}\;+\;\beta_{4}{\text{Region}}_{i}\;+\;\beta_{5}{\text{Migration}}_{i}\;+\;\text{offset}\;\left(\text{population}\right)$$

The annual count of drug-resistant cases was the dependent variable, modelled with a log (population)^22^ offset; independent variables were period, sex, age group, region, and migration status. Reference categories were set as 2009–2014, representing a stable pre-implementation interval with consistent laboratory workflows and high data completeness (2003–2008 had lower volumes and more missing covariates). Additional reference categories were female sex, ≥65 years, residence in the Metropolitan Region, and migrant status, in line with routine surveillance reporting. Prior treatment status was not included as an adjustment variable to avoid conditioning on a mediator and reduce collinearity; HIV status was not included due to substantial missingness and non-systematic capture of HIV-negative status. Results were reported as incidence rate ratios (IRRs). Statistical analyses were conducted in Stata v17 (StataCorp, College Station, TX) at a significance level of 0.05 with 95% confidence intervals (CIs).

### Ethical statement

Ethics approval was not required for this retrospective analysis of anonymised surveillance data, conducted under PROCET and in accordance with the national technical standard for TB control, which mandates the National Reference Laboratory to perform DST.^18^

## RESULTS

During the period 2003–2020, the ISP processed 44,800 culture-confirmed MTB isolates for DST. By period, totals were 15,540 (2003–2008), 13,310 (2009–2014), and 15,950 (2015–2020); of these, 15,475, 13,109, and 15,305 were susceptible to both INH and RIF, respectively. Annual denominators (total processed, INH/RIF-susceptible, and resistant counts with phenotype breakdown) are provided in Supplementary Data Table S1, together with the annual proportions of first-line resistance.

**Figure 1.**
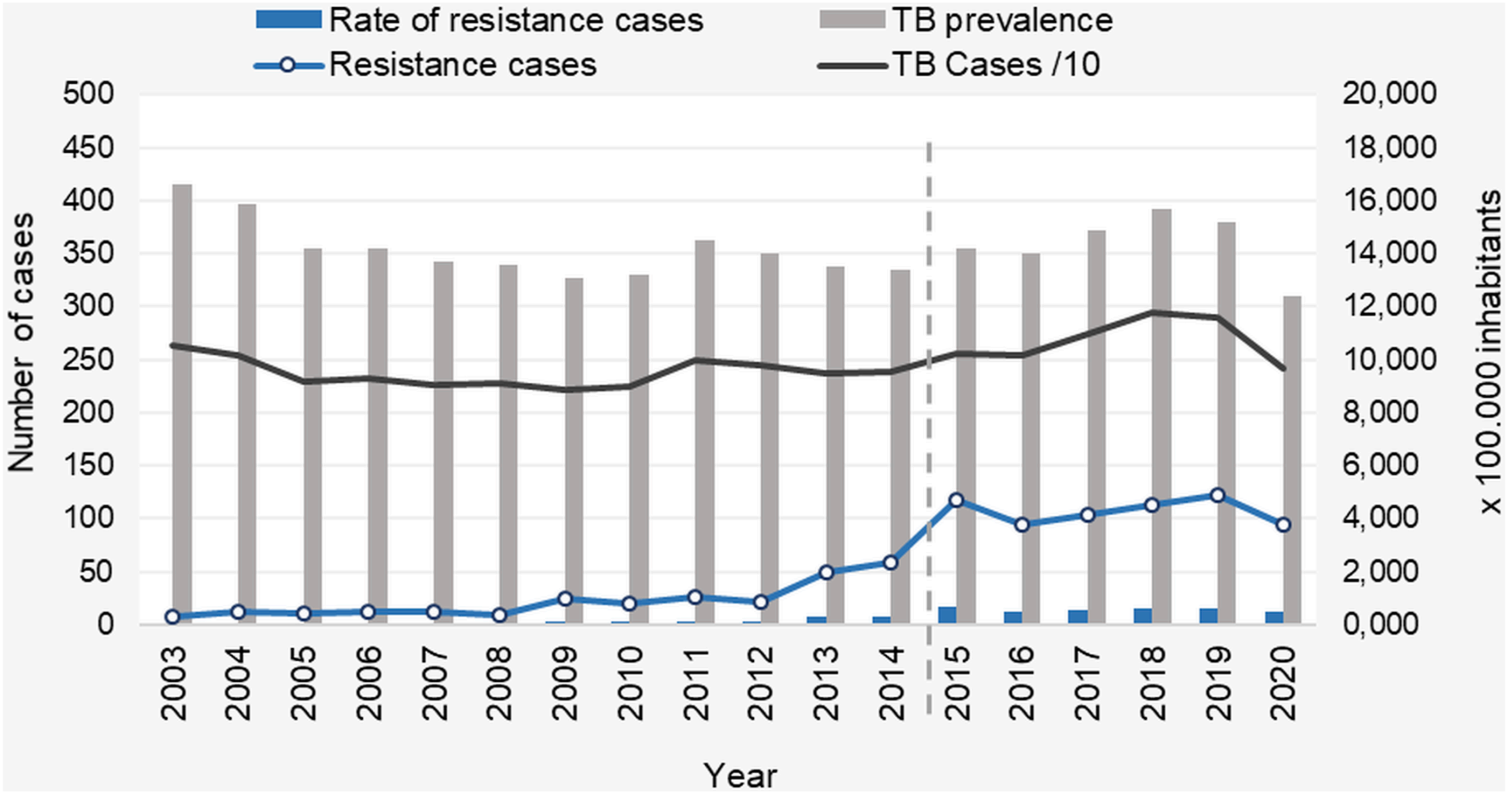
Resistance and TB analysis in Chile. Cases and TB rates, along with resistance cases from 2003 to 2020. Prevalence and total case numbers were obtained from the annual reports of the National Tuberculosis Program (DIPRECE – MINSAL). A dotted line marks the beginning of universal surveillance.

**Figure 2.**
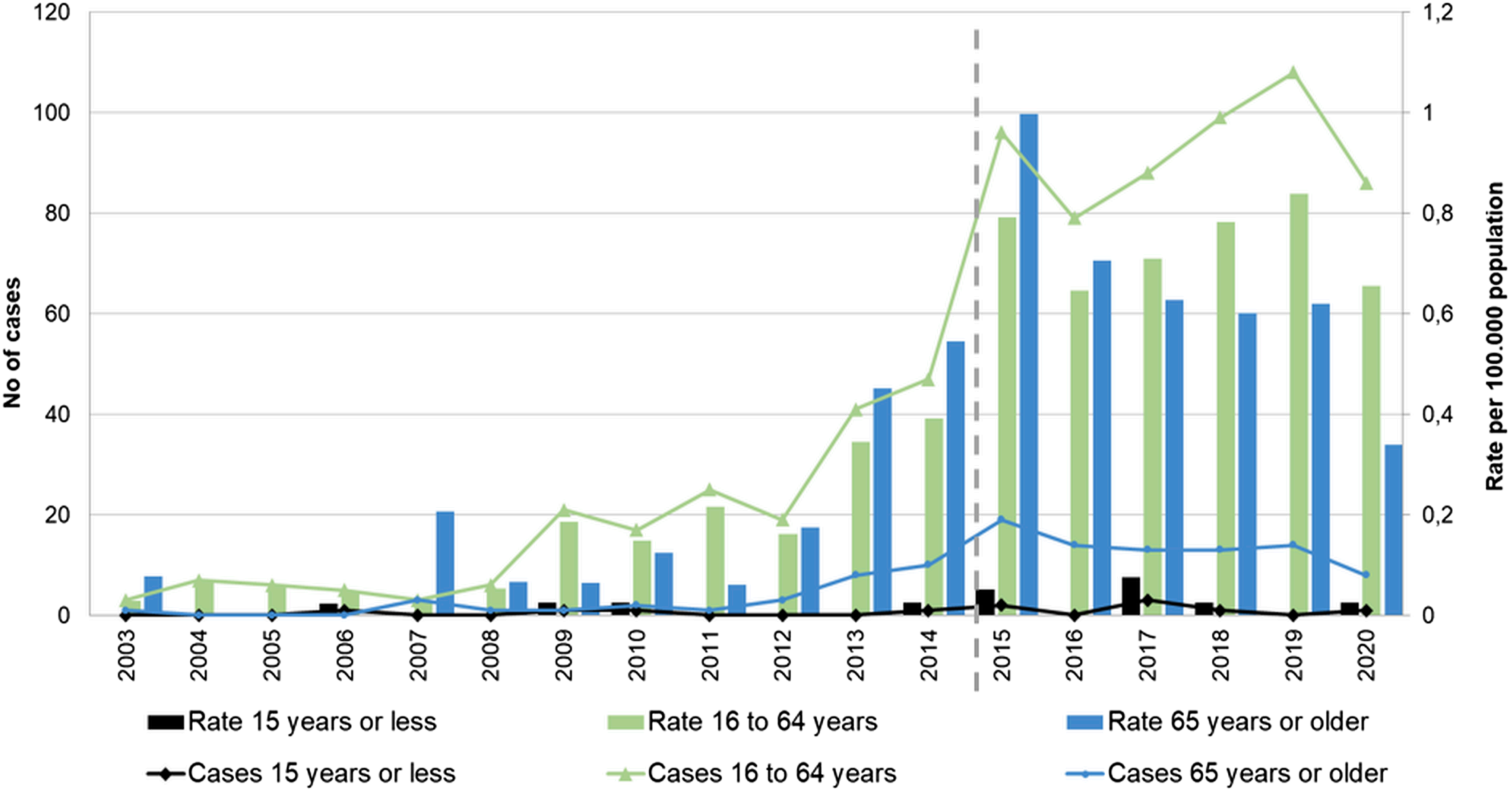
Distribution of TB cases by age group. Annual number of cases and incidence rates per 100,000 population, stratified into three age groups: ≤15 years, 16–64 years, and ≥65 years.

**Figure 3.**
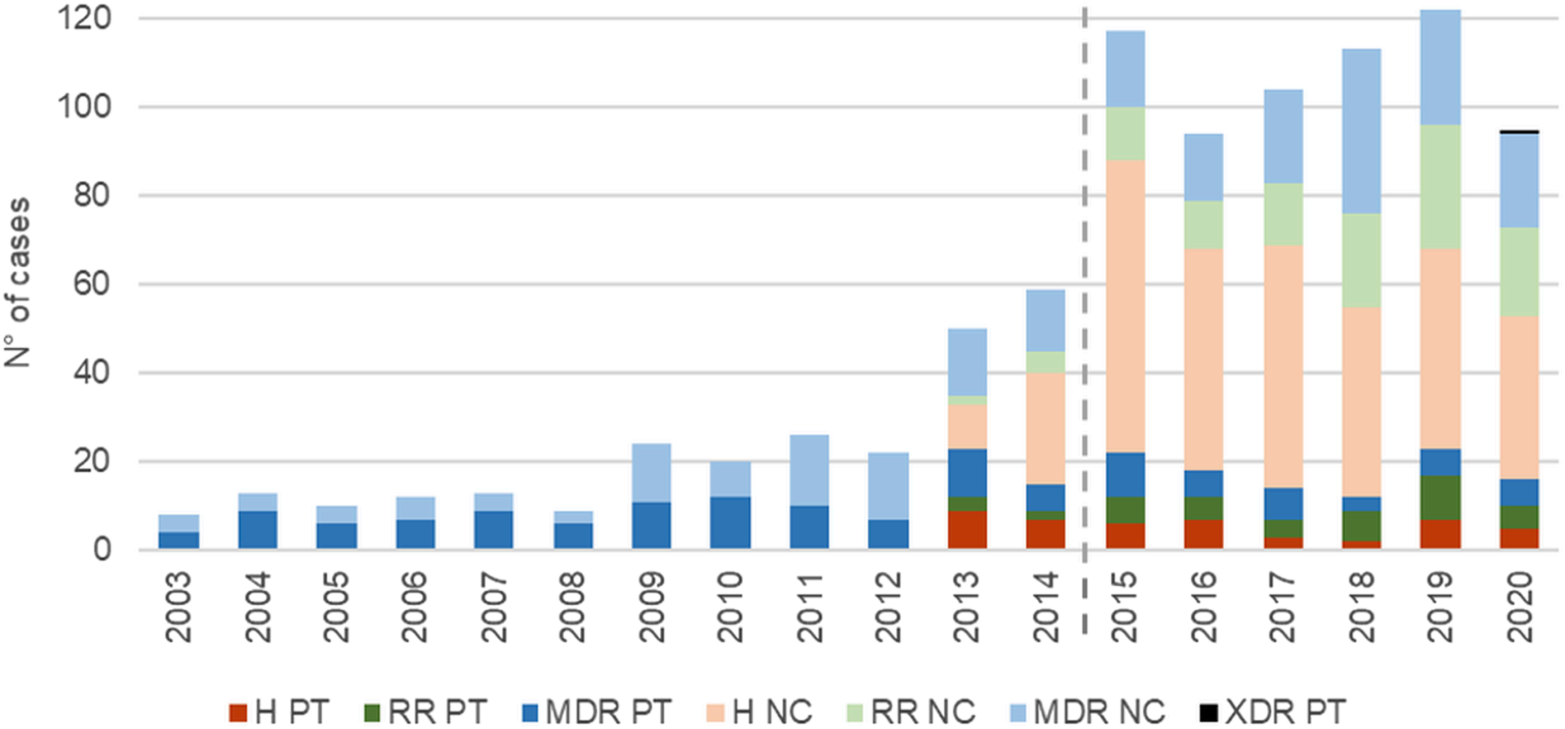
Previous treatment history and resistance profiles in TB cases. Relationship between previous treatment history and resistance profiles among TB cases. Previous treatment was classified as new cases (NC) or previously treated (PT). RR = rifampicin-resistance; MDR = multidrug resistance; XDR = extensively drug resistance.

**Figure 4.**
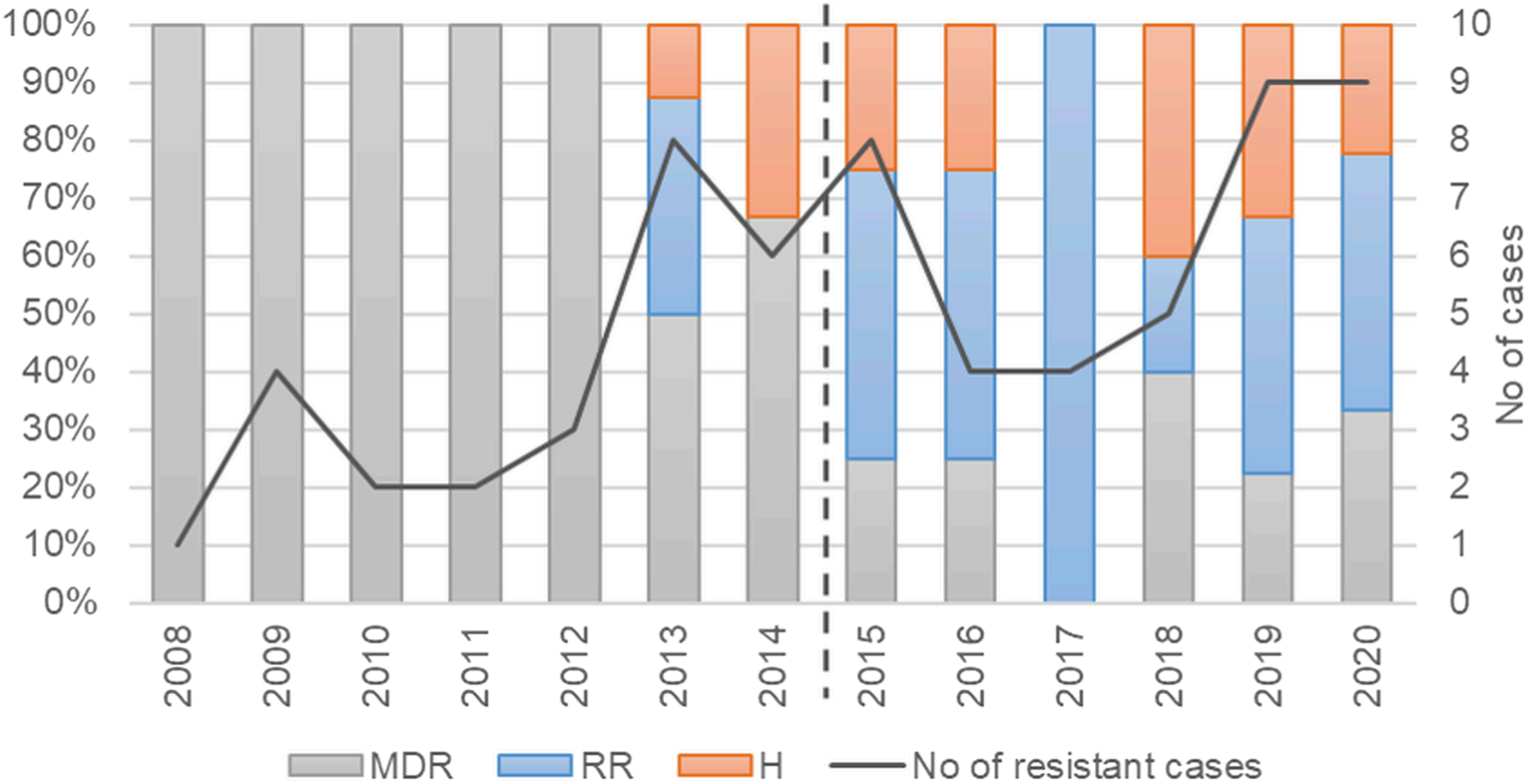
Resistance profiles among HIV-positive TB cases. Resistance profiles (monoresistant, MDR, RIF, and XDR) among HIV-positive patients. MDR = multidrug resistance; RIF = rifampicin; XDR = extensively drug resistance; RR = rifampicin resistance.

In total, 911 resistant isolates were identified: 377 INH monoresistance, 155 RIF monoresistance, 378 MDR, and 1 XDR. These corresponded to 0.42%, 1.51%, and 4.04% of all processed isolates in 2003–2008, 2009–2014, and 2015–2020, respectively (see Supplementary Data Table S3). Demographic and clinical characteristics of the study population are summarised by surveillance period in Supplementary Data Table S2. The number of resistance cases and TB prevalence rates in Chile were analysed and are shown in Figure 1. The distribution of TB cases by age group is shown in Figure 2. The distribution of resistance profiles by treatment history is shown in Figure 3. Resistance profiles among HIV-positive cases are presented in Figure 4.

### Negative binomial regression

Compared with 2009–2014 (reference), detection rates were lower in 2003–2008 (IRR = 0.323; 95% CI: 0.193–0.539; *P* < 0.001) and higher in 2015–2020 (IRR = 3.592; 95% CI: 2.611–4.942; *P* < 0.001). Rates were higher in men than women (IRR 2.429; 95% CI: 1.836–3.215; *P* < 0.001). Compared with those aged ≥65 years, rates were higher in 16–64-year-olds (IRR 5.827; 95% CI: 4.159–8.164; *P* < 0.001) and lower in ≤15-year-olds (IRR 0.217; 95% CI: 0.100–0.474; *P* < 0.001). Relative to the central macrozone, rates were lower in the north (IRR 0.322; 95% CI: 0.214–0.483), north central (IRR 0.316; 95% CI: 0.211–0.473), south central (IRR 0.247; 95% CI: 0.161–0.380), and south (IRR 0.191; 95% CI: 0.121–0.301); all *P* < 0.001. Foreign-born patients had lower rates than Chilean patients (IRR 0.342; 95% CI: 0.246–0.475; *P* < 0.001). Overdispersion was present (α = 0.153; 95% CI: 0.082–0.287), supporting the negative binomial specification. Full adjusted estimates are shown in Table.

**Table.** Negative binomial regression model used to assess the impact of universal surveillance implementation.

	IRR	Std. err.	z	*P* value	(95% CI)	
					LCI	UCI
Policy						
2003–2008	0.323	0.084	−4.32	0	0.193	0.539
2009–2014	Ref					
2015–2020	3.592	0.585	7.86	0	2.611	4.942
Sex						
Male	2.429	0.347	6.21	0	1.836	3.215
Female	Ref					
Age						
15 years or less	0.217	0.087	−3.83	0	0.1	0.474
16–64 years	5.827	1.002	10.25	0	4.159	8.164
65 years or older	Ref					
Macrozone of residence						
North	0.322	0.067	−5.46	0	0.214	0.483
North Central	0.316	0.065	−5.59	0	0.211	0.473
South Central	0.247	0.054	−6.38	0	0.161	0.38
South	0.191	0.044	−7.11	0	0.121	0.301
Central	Ref					
Migration status						
Foreign	0.342	0.057	−6.39	0	0.246	0.475
Chilean	Ref					
cte.	2.613	0.595	4.22	0	1.672	4.082
ln(α)	−1.878	0.32			−2.505	−1.25
α	0.153	0.049			0.082	0.287

Samples with missing age or nationality information were excluded from the analyses related to these variables. Results are presented as incidence rate ratios (IRR) with 95% confidence intervals (CI).

LCI = lower confidence interval; UCI = upper confidence interval.

## DISCUSSION

The results of this study show a stepwise increase in first-line drug-resistance detection across three surveillance periods, with higher detection in 2009–2014 than 2003–2008 and a larger increase in 2015–2020 than 2009–2014. This reflects increased detection yield following two sequential changes: the progressive adoption of molecular resistance testing (LPA as the primary assay from 2011) and the subsequent implementation of systematic, universal first-line INH/RIF screening from mid-2014 onwards, replacing earlier selective testing. The WHO estimates that up to 62% of global drug-resistant TB cases are undiagnosed.^23^ The marked rise in detection from 2015 supports the hypothesis of substantial underdiagnosis during the earlier selective testing phase.

Demographically, most cases occurred in men, aligning with the higher TB burden in men.^23^ The 16–64-year group was most affected, likely reflecting greater mobility and social interaction. Older adults (≥65 years) represented a smaller proportion of cases, but their higher vulnerability to severe TB due to age-related immune decline supports targeted prevention strategies in this group (Figure 2).

Geographically, the central macrozone consistently had the highest case counts, likely due to higher population density. In some years, the northern region showed higher resistance rates (Supplementary Data Table S3), which should be interpreted with caution given temporal variation in testing coverage. Neighbouring countries such as Peru and Bolivia report higher TB incidence and resistance, possibly contributing to drug resistance in northern Chile.^15^ By contrast, the southern region reported lower resistance rates, likely due to lower population density and reduced mobility.

Regarding patient nationality, most resistant cases were found in Chilean individuals. After 2015, an increase was observed among Chilean and foreign patients, and the majority of foreign-born cases came from Peru, Haiti, Bolivia, and Colombia (Supplementary Data Figure S1), countries with higher TB prevalence than Chile.^15^ These findings underscore the importance of sustained surveillance in migrant populations and regional cooperation to prevent the spread of drug-resistant TB.

Four resistance profiles were identified: INH, RIF, MDR, and XDR, with an increase in INH-resistant detections after 2013 (Supplementary Data Figure S2). Because first-line screening targeted INH and RIF only, resistance to other drugs (e.g., ethambutol and pyrazinamide) was mainly detected after negative/invalid LPA, or via MGIT based on clinical or epidemiological indications. Accordingly, the reported profiles reflect the detection strategy, and less frequent patterns outside this panel may have been under-ascertained. Nevertheless, these four categories are the most directly actionable for programmatic management, informing first-line treatment decisions and routine drug-resistance surveillance.

MDR-TB cases fluctuated, peaking in 2018 and then slightly declining. Although XDR-TB cases were rare, one was identified in 2020, highlighting the need for ongoing monitoring and control strategies to prevent its spread. To explore the relationship between resistance and treatment history, cases were grouped into ‘new’ (no prior treatment) and ‘previously treated’ categories (Supplementary Data Figure S3). Although treatment regimens are standardised nationally, individual-level regimen and outcome data were unavailable, limiting interpretation by treatment history and precluding analyses of time to effective therapy, culture conversion, or relapse. After adoption of universal surveillance, detections among new resistant cases increased relative to previously treated (Figure 3); however, given expanded screening coverage and inclusion criteria, these patterns are best interpreted as improved detection at diagnosis rather than evidence of a shift in transmission. These trends are consistent with community transmission; however, this cannot be confirmed without genomic or contact-tracing data. Programmatically, the findings support maintaining broad first-line resistance screening at diagnosis and timely contact-tracing to limit onwards spread.

Immunocompromised individuals, particularly those living with HIV, are at higher risk of TB.^9^ From 2008 onwards, drug-resistant TB cases increased among people with HIV; however, interpretation is limited by the absence of HIV-associated resistance between 2003 and 2007, which likely reflects underreporting or incomplete data capture rather than true absence of cases (Supplementary Data Figure S4). Despite access to antiretroviral therapy in Chile, HIV/TB co-infected individuals remain a high-risk group requiring integrated care strategies.^24^ Resistance patterns in this group varied, with sporadic cases during 2008–2012 and a gradual rise in MDR and RIF resistance from 2013 onwards (Figure 4).

A negative binomial regression model confirmed that detected drug-resistant TB was higher after national scale-up and lower before it, relative to 2009–2014 (Table), supporting interpretation of the post-2014 rise as improved detection under expanded screening rather than an increased incidence. Sex, age, macrozone, and nationality also influenced detected resistance trends: men and adults aged 16–64 had higher incidence rates than women and older adults, consistent with global patterns and likely driven by increased mobility and social interaction. Metropolitan Center showed the highest resistance incidence, whereas other macrozones showed lower rates, likely reflecting differences in population density and transmission dynamics.

Foreign-born individuals constituted a minority of cases and had a lower incidence of drug-resistant TB than Chilean-born individuals. However, this result may be influenced by small sample size and incomplete nationality recording in historical data, which reinforces the need to maintain targeted surveillance in migrant populations, particularly given the higher TB burden in many of their countries of origin.

## CONCLUSION

The model results suggest that universal surveillance has substantially improved detection of drug-resistant TB in Chile. Nevertheless, persistent disparities by sex, age, geographic location, and nationality highlight the need for targeted interventions and continued refinement of TB control strategies for high-risk and underserved populations to sustain progress against drug-resistant TB and align with global elimination goals.

## Supplementary Material
