# Supplementary material for: Resistance to anti-TB drugs: evaluation of universal resistance surveillance implementation in Chile
Source: IJTLD Open. 2026 Jun 15;3(6):384–90. doi: 10.5588/ijtldopen.25.0507 (PMC13268052; doi:10.5588/ijtldopen.25.0507)

Supplementary Tables

Table S1. Annual counts of processed isolates, INH/RIF-susceptible, and resistant phenotypes (2003–2020).

Year	Total processed isolates (n)	INH/RIF susceptible (n)	Resistance				Resistant total	Resistant among processed (%)
			INH	RIF	MDR	XDR		
2003	2,908	2,900	0	0	8	0	8	0.28%
2004	2,793	2,780	0	0	13	0	13	0.47%
2005	2,546	2,536	0	0	10	0	10	0.39%
2006	2,557	2,545	0	0	12	0	12	0.47%
2007	2,462	2,449	0	0	13	0	13	0.53%
2008	2,274	2,265	0	0	9	0	9	0.40%
2009	2,218	2,194	0	0	24	0	24	1.08%
2010	2,254	2,234	0	0	20	0	20	0.89%
2011	2,262	2,236	0	0	26	0	26	1.15%
2012	2,201	2,179	0	0	22	0	22	1.00%
2013	2,190	2,140	19	5	26	0	50	2.28%
2014	2,185	2,126	32	7	20	0	59	2.70%
2015	2,385	2,268	72	18	27	0	117	4.91%
2016	2,548	2,454	57	16	21	0	94	3.69%
2017	2,740	2,636	58	18	28	0	104	3.80%
2018	2,945	2,832	45	28	40	0	113	3.84%
2019	2,912	2,790	52	38	32	0	122	4.19%
2020	2,420	2,325	42	25	27	1	95	3.93%
**Total**	**44,800**	**43,889**	**377**	**155**	**378**	**1**	**911**	**2.03%**
Period summary								
2003–2008	15,540	15,475	0	0	65	0	65	0.42%
2009–2014	13,310	13,109	51	12	138	0	201	1.51%
2015–2020	15,950	15,305	326	143	175	1	645	4.04%

Horizontal rule denotes the boundary between pre-implementation (≤2014) and post-implementation periods of universal INH/RIF screening.

Table S2. Demographic and Clinical Characteristics of TB cases analysed between 2003 and 2020.

	Before (2003 - 2008) n= 65		Before (2009- 2014) n= 201		After (2015 - 2020) n= 645		Total (2003 - 2020) n= 911		% of cases between 2015 and 2020 relative to the total number of cases (2003-2020)
	cases	%	cases	%	cases	%	cases	%	%
**Demographic characteristics**									
**Sex**									
Female	13	20.00%	54	26.87%	184	28.53%	251	27.55%	73.31
Male	52	80.00%	147	73.13%	461	71.47%	660	72.45%	69.85
**Age**									
15 years or less	1	1.54%	3	1.49%	7	1.09%	11	1.21%	63.64
16 to 64 years	30	46.15%	170	84.58%	556	86.20%	756	82.99%	73.54
65 years or more	5	7.69%	25	12.44%	81	12.56%	111	12.18%	72.97
No information	29	44.62%	3	1.49%	1	0.16%	33	3.62%	3.03
**Macrozone**									
North	4	6.15%	30	14.93%	85	13.18%	119	13.06%	71.43
North central	10	15.38%	29	14.43%	79	12.25%	118	12.95%	66.95
Central	38	58.46%	101	50.25%	351	54.42%	490	53.79%	71.63
South central	9	13.85%	30	14.93%	80	12.40%	119	13.06%	67.23
South	4	6.15%	11	5.47%	50	7.75%	65	7.14%	76.92
**Migration**									
Chilean	32	49.23%	124	61.69%	433	67.13%	589	64.65%	73.51
Foreigner*	6	9.23%	25	12.44%	192	29.77%	223	24.48%	86.1
No information	27	41.54%	52	25.87%	20	3.10%	99	10.87%	20.2

	Before (2003 - 2008) n= 65		Before (2009- 2014) n= 201		After (2015 - 2020) n= 645		Total (2003 - 2020) n= 911		% of cases between 2015 and 2020 relative to the total number of cases (2003-2020)
	cases	%	cases	%	cases	%	cases	%	%
**Clinical characteristics**									
**Resistance**									
H	0	0.00%	51	25.37%	326	50.54%	377	41.38%	86.47
MDR, RR, XDR**	65	100%	150	74.63%	319	49.46%	534	58.62%	59.74
**Treatment**									
Treated	41	63.08%	78	38.81%	106	16.43%	225	24.70%	47.11
New cases	24	36.92%	123	61.19%	539	83.57%	686	75.30%	78.57
**VIH**									
Non-specific	64	98.46%	176	87.56%	606	93.95%	846	92.86%	71.63
Positive	1	1.54%	25	12.44%	39	6.05%	65	7.14%	60

Data was analysed in three periods: two 5-year periods before universal surveillance, and one 5-year period after its implementation. Chile was divided into macrozones: North (Arica to Antofagasta), North central (Atacama to Valparaíso), Central (Metropolitana y O'Higgins), South central (Maule a Bío-Bío), and South (La Araucanía a Magallanes).

* Before 2009, 100% of foreign cases were of Peruvian nationality; between 2009 and 2014, 84.0% were Peruvians, while the remaining 16% were from other nationalities. From 2015 to 2020, 54.2% of the foreign cases were Peruvian, 16.6% were Haitian, and the remaining 30.2% were from other nationalities.

** Only one case of XDR resistance was reported, corresponding to the year 2020.

Table S3. Annual number and rate of resistance cases by macrozone.

Year	Cases North	Rate North	Cases North Central	Rate North Central	Cases Metropolitan Central	Rate Metropolitan Central	Cases South Central	Rate South Central	Cases South	Rate South
2003	0	0.0	1	0.04	3	0.042	3	0.103	1	0.044
2004	1	0.103	2	0.078	7	0.096	2	0.068	1	0.044
2005	1	0.101	2	0.078	4	0.054	2	0.068	1	0.043
2006	2	0.199	2	0.077	6	0.081	2	0.068	0	0.0
2007	0	0.0	2	0.076	11	0.146	0	0.0	0	0.0
2008	0	0.0	1	0.037	7	0.092	0	0.0	1	0.042
2009	4	0.379	6	0.222	10	0.13	2	0.066	2	0.084
2010	4	0.373	2	0.073	7	0.09	6	0.196	1	0.041
2011	3	0.275	4	0.144	14	0.178	5	0.162	0	0.0
2012	1	0.09	7	0.249	11	0.138	2	0.064	1	0.041
2013	11	0.976	5	0.176	23	0.286	7	0.224	4	0.162
2014	7	0.611	5	0.174	36	0.443	8	0.254	3	0.121
2015	16	1.374	15	0.515	53	0.645	24	0.755	9	0.359
2016	13	1.099	11	0.373	48	0.577	11	0.343	11	0.436
2017	13	1.081	16	0.536	59	0.697	8	0.248	8	0.315
2018	18	1.449	14	0.463	60	0.692	14	0.43	7	0.274
2019	13	1.012	14	0.456	73	0.821	14	0.427	8	0.31
2020	12	0.904	9	0.289	58	0.636	9	0.272	7	0.27
Total	119		118		490		119		65	

Number of resistant TB cases and incidence rate per 100.000 population, stratified by year and macrozone. A horizontal rule marks the start of the period following the implementation of resistance surveillance.

Supplementary Figures

Figure S1. *Distribution of foreign-born tuberculosis cases by nationality.*

Bars represent the number of foreign-born tuberculosis cases for each nationality. There were 99 cases for which nationality information was not available.

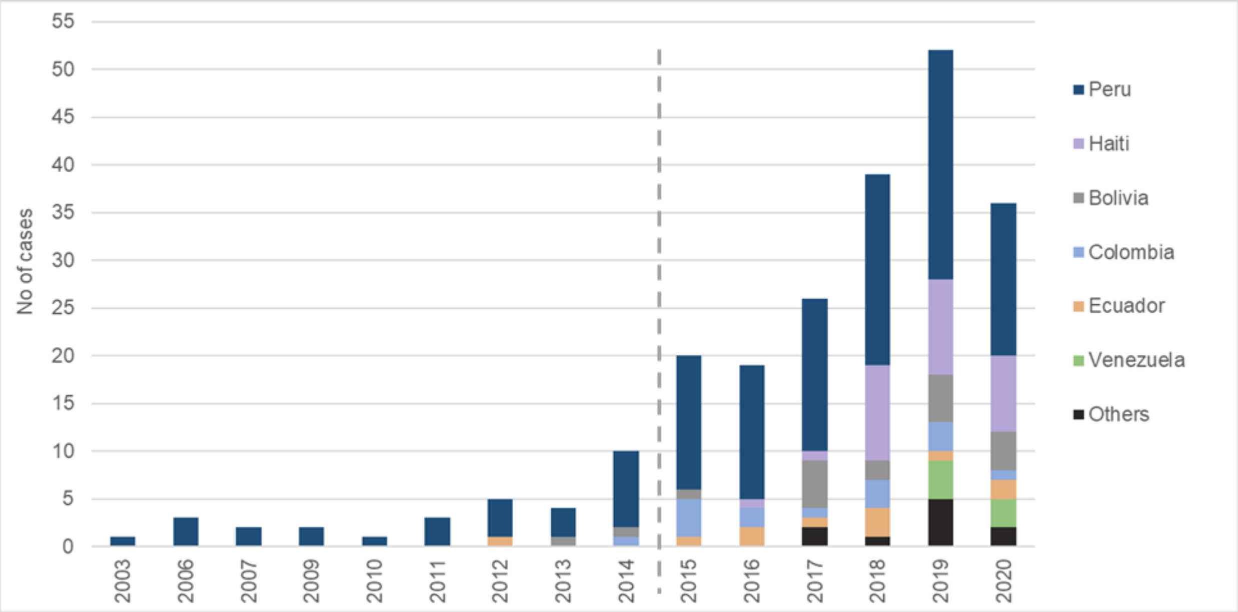

Figure S2. *Distribution of resistance profiles in tuberculosis cases.*

Annual number of cases with H and RR/MDR/XDR profiles; dashed line indicates the start of universal resistance surveillance.

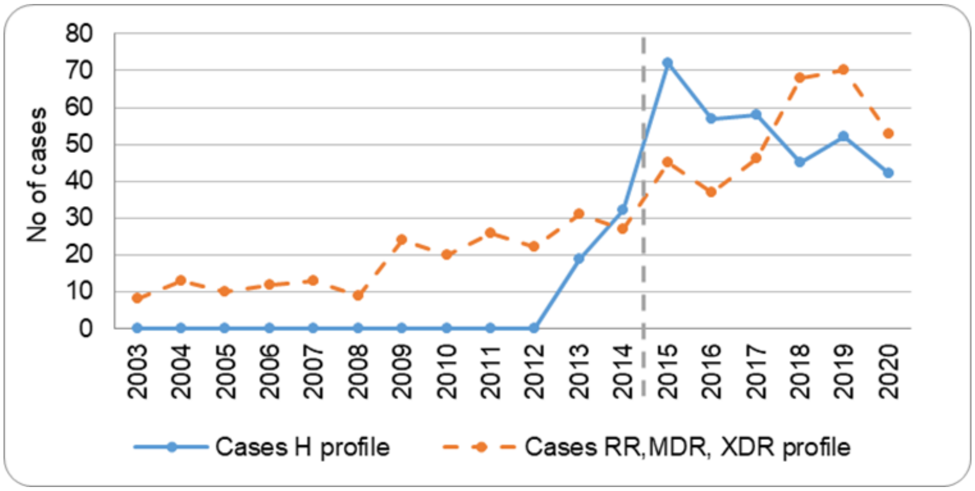

Figure S3. *Distribution of resistance profiles in tuberculosis cases by treatment history.*

Annual number of cases among new cases (NC) and previously treated (PT) patients; the vertical dashed line indicates the start of universal resistance surveillance.

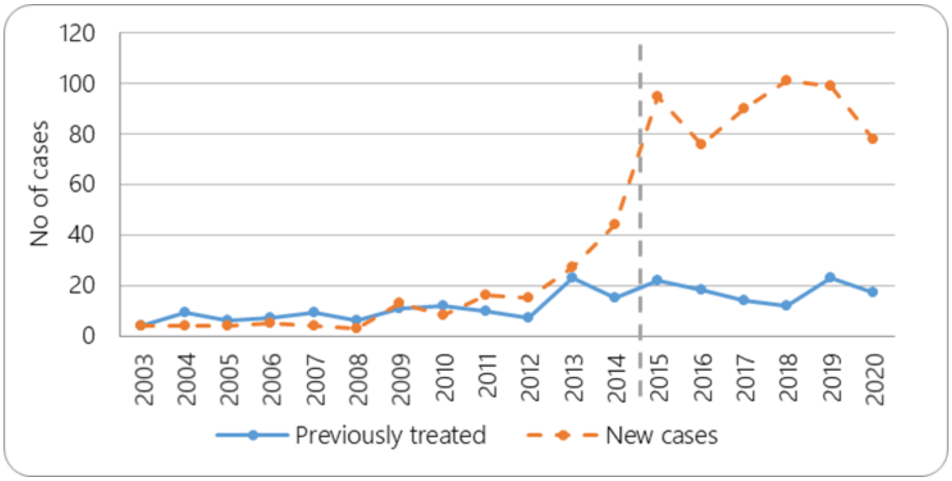

Figure S4. *Annual number of drug-resistant TB cases by recorded HIV status, 2003–2020.*

HIV status categories are “positive” and “not specified” (missing/unreported HIV status, not HIV negative). The dashed line indicates the start of universal first-line resistance screening.

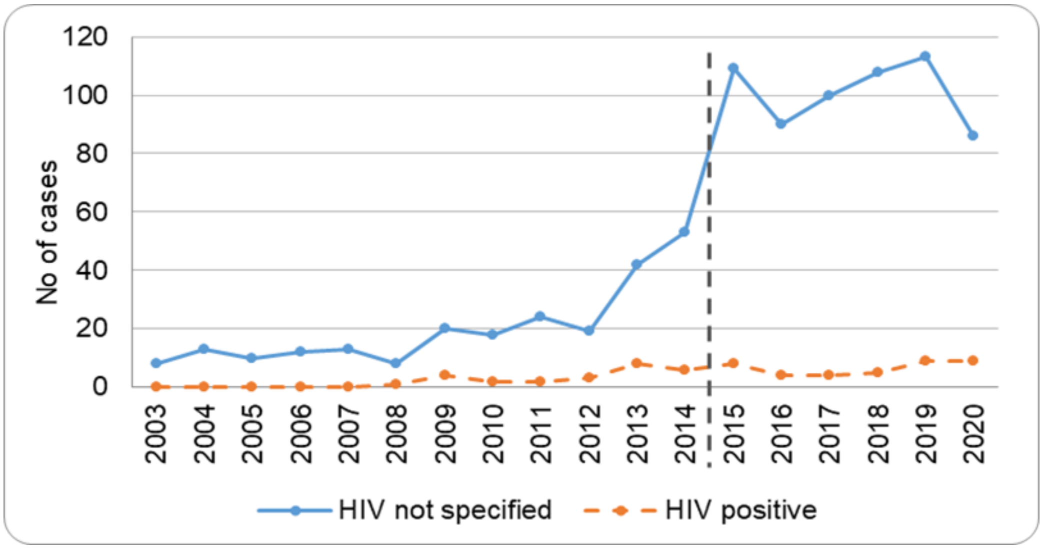

Supplement: Supplementary file 1 [file ijtldopen25-0507_supplementarydata1.pdf]
